# Bilateral myelin oligodendrocyte glycoprotein antibody-associated disease after cataract surgery

**DOI:** 10.1016/j.ajoc.2026.102518

**Published:** 2026-01-24

**Authors:** Benjamin Besse, Amélie Dos Santos, Mathieu Mille, Francois Bobin, Hugo Schultz, Aline Berthomet, Léo Niemerich, Anna Muszalski, Favia Gianrocco, Nicolas Leveziel

**Affiliations:** a Department of Ophthalmology, CHU Poitiers, France; b Department of Neurology, CHU Poitiers, France; c Polyclinique de Poitiers, France; d Department of Neurophysiology, CHU Poitiers, France; e Department of Radiology, CHU Poitiers, France; f INSERM 1084, Poitiers, France; g CNRS I3M, Poitiers, France

**Keywords:** AION, Cataract, MOGAD, Myelin oligodendrocyte glycoprotein antibody-associated disease, NAION, Optic neuritis

## Abstract

**Purpose:**

To report an unusual presentation of bilateral myelin oligodendrocyte glycoprotein antibody-associated disease (MOGAD) associated with optic neuritis occurring during the early post-operative cataract surgical period.

**Methods:**

This was an observational case report.

**Results:**

A 67-year-old Caucasian female presented with right eye visual acuity decrease occurring five days after bilateral cataract surgery. She also reported moderate pain associated with eye movements. While no inflammation could be observed, bilateral optic disc edema was observed on fundus examinations. Fluorescein angiography showed leakage of dye at late phases at the optic nerves, with no retinal vasculitis or choroidal ischemia on either eye. Orbit and brain magnetic resonance imaging showed bilateral optic perineuritis. Cerebrospinal fluid analysis was normal, as was the opening pressure (<25 cm of water) measured during lumbar puncture. Blood testing revealed MOGAD Immunoglobulin G (IgG) autoantibodies at a titer >1:100.

**Conclusion:**

This report describes an atypical presentation of MOGAD associated with bilateral optic neuritis occurring a few days after cataract surgery.

## 1 Introduction

The main causes of visual acuity (VA) decrease during early post-operative period after cataract surgery include endophthalmitis, corneal decompensation and Irvin Gass syndrome. These diagnoses are usually ruled out after slit lamp examination, fundus examination and optical coherence tomography (OCT) scans centered on the macula.

Optic disc edema is commonly observed in non-arteritic anterior ischemic optic neuropathy (NAION), arteritic anterior ischemic optic neuropathy (AION), optic neuritis and cases of orbital lesions.

For NAION, crowded optic discs and the post-operative period after cataract surgery have been described as common risk factors.^1^^,^^2^ The other main risk factors, aside from age and male sex, are obstructive sleep apnea, metabolic syndrome and cardiovascular disease.^3^^,^^4^

Causes of optic neuritis include mainly myelin oligodendrocyte glycoprotein antibody-associated disease (MOGAD), infectious diseases, immunomodulated disorders (i.e. granulomatosis with polyangiitis and sarcoidosis), and neoplasia (myeloma, leukemia, lymphoma).^5^

We report a case of bilateral optic neuritis occurring one week after uncomplicated cataract surgery in a woman aged 67 years with no history of cardiovascular or inflammatory disease.

## 2 Case report

**Fig. 1:**
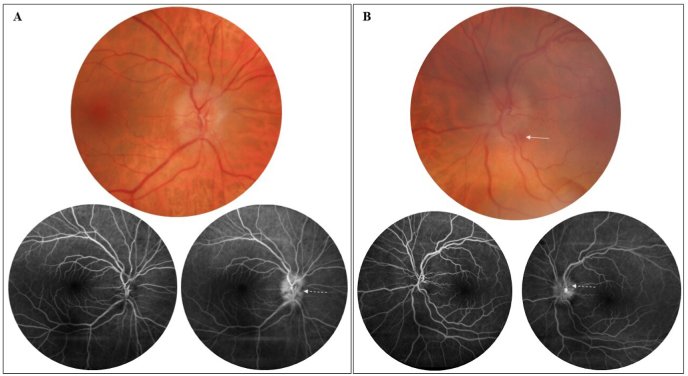
Fundus photographs and fluorescein angiograms of the right (A) and left (B) eyes of a 67-year-old woman who presented with visual acuity decrease in the right eye and bilateral moderate ocular pains occurring with eye movements five days after uncomplicated bilateral cataract surgery. Diffuse optic disc edema in both eyes, with peripapillary retinal hemorrhage inferotemporal to the optic disc in the left eye (arrow). On fluorescein angiography, a progressive staining and leakage of the dye was observed on the optic nerve in both eyes during late phases of the angiogram (shown on the right), more pronounced in the right eye (dashed arrows).

**Fig. 2:**
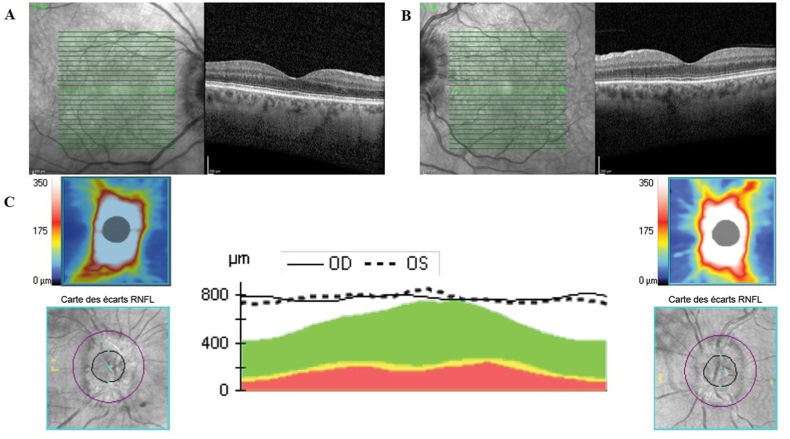
Optical coherence tomography centered on the fovea in the right eye (A) and left eye (B) performed at presentation of a 67-year-old woman with visual acuity decrease in the right eye and bilateral moderate ocular pains occurring with eye movements five days after uncomplicated bilateral cataract surgery. There was no macular edema (A, B). Optic disc cube in the right and left eyes (C) showing diffuse increase of retinal nerve fiber layers in both eyes.

A 67-year-old woman presented with VA decrease in the right eye occurring five days after uncomplicated cataract surgery, while cataract surgery on the left eye had been performed four days before surgery of the right eye. Prior to surgery, her refractive status in spherical equivalent was +0.75 and + 2.25 diopters in the right and left eyes, respectively, and cup-to-disc ratio measured on OCT scan was 0.3 in both eyes. At presentation, she reported bilateral ocular pains increased by ocular movements that appeared the day before. Best-corrected VA was measured at 20/200 and 20/20 in the right and left eyes, respectively. On slit lamp examination, there was no anterior segment inflammation, but on fundus examination there was bilateral optic nerve edema with a peripapillary hemorrhage in the left eye (Fig. 1). On fluorescein angiography (FA), neither optic disc ischemia nor choroidal ischemia was observed. A progressive staining and leakage of the dye on the optic nerve was observed during late phases of the angiogram on both eyes (Fig. 1). A blood test for measurement of C-Reactive protein (CRP) and erythrocyte sedimentation rate (ESR) levels and FA were performed immediately to rule out AION associated with giant cell arteritis. Macular OCT scans did not show macular edema, while bilateral diffuse increase of retinal nerve fiber layers was observed on optic disc OCT scan (Fig. 2).

**Fig. 3:**
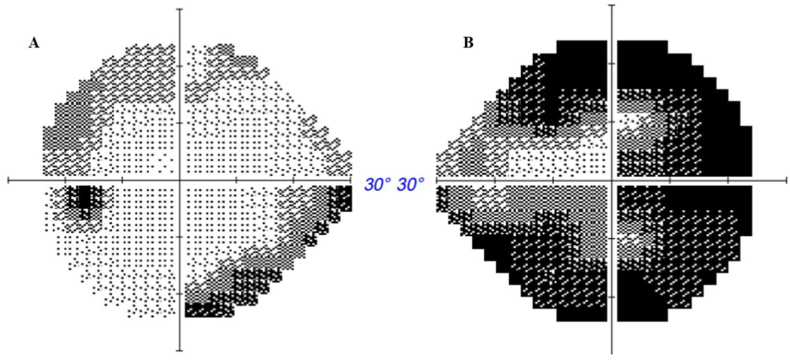
30-2 Humphrey visual field the day after presentation of a 67-year-old woman with visual acuity decrease in the right eye and bilateral moderate ocular pains occurring with eye movements five days after uncomplicated bilateral cataract surgery. In the left eye: superior arcuate and inferior nasal depressions (A). In the right eye: marked generalized depression, predominantly temporal (B).

**Fig. 4:**
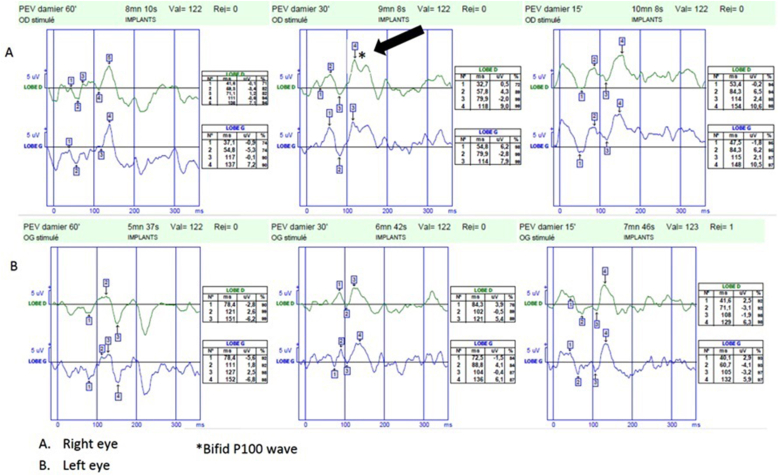
Visual evoked potentials performed three weeks after presentation of a 67-year-old woman with visual acuity decrease in the right eye and bilateral moderate ocular pains occurring with eye movements five days after uncomplicated bilateral cataract surgery. Delayed P100 latency (arrowhead) with no reduced amplitude, with bifid wave (∗) on the right eye (A for right eye, B for left eye).

On ancillary investigation, an arcuate visual field defect with peripheral constriction, predominant on the temporal part, was observed in the right eye, and a less pronounced arcuate visual field defect was observed in the left eye on 30-2 visual field (Fig. 3), while visual evoked potentials showed delayed P100 latencies with no reduced amplitudes and bifid waves on the right eye (Fig. 4).

**Fig. 5:**
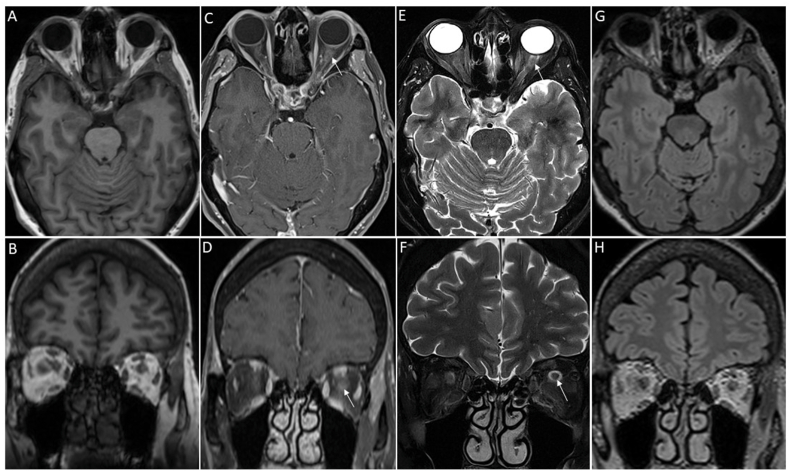
Brain and orbital magnetic resonance imaging (MRI) performed the day of presentation of a 67-year-old woman who had a visual acuity decrease in the right eye associated with bilateral moderate ocular pains occurring with eye movements five days after uncomplicated bilateral cataract surgery. Non-contrast axial (A) and coronal (B) T1-weighted images showing no relevant anomalies. Post-contrast fat-saturated axial (C) and coronal (D) T1-weighted images. There is tortuosity of the right optic nerve. Thickening and contrast enhancement of the optic nerves were observed, predominantly on the left eye (arrows). Axial (E) and coronal (F) fat-saturated T2-weighted images. The left optic nerve shows thickening and hyperintensity (E, arrow). There is dilatation of the subarachnoid spaces of the optic nerve sheaths, predominantly on the left (F, arrow). Axial (G) and coronal (H) T2 Fluid-Attenuated Inversion Recovery (FLAIR) showing no relevant anomalies.

On orbit and brain magnetic resonance imaging (MRI) performed the same day, optic perineuritis with optic nerve sheath inflammation and enhancement was observed on the axial and coronal fat-saturated longitudinal relaxation time (T1) and fat-saturated spin–spin relaxation time (T2) sections, mainly in the left eye (Fig. 5).

The patient was admitted to the neurology unit for further investigation, including a lumbar puncture and other blood tests targeting potential infectious diseases (tuberculosis, syphilis, borrelia burgdorferi, herpes simplex, cytomegalovirus, Epstein-Barr virus and Chlamydia pneumoniae) and autoimmune disorders by searching for anti-nuclear autoantibodies, anti-neutrophil cytoplasmic antibodies (ANCA), anti-water channel aquaporin 4 (AQ4) and MOGAD Immunoglobulin G (IgG) autoantibodies. Cerebrospinal fluid analysis was normal, as was the measurement of opening pressure, which was below 25 cm of water during lumbar puncture. Serum MOGAD IgG antibodies were positive, with titer >1:100, confirming the diagnosis of MOGAD-associated optic neuritis.

The patient was treated with intravenous methylprednisolone 500 mg/day for 3 days, and then switched to oral prednisolone 60 mg per day for 1 month, with progressively decreasing doses of corticosteroid over a period of 6 months. No recurrence, or other neurological events were reported during the follow-up period of 9 months.

Right eye best-corrected VA reverted to 20/30 three days after corticoid initiation and best-corrected VA was measured at 20/20 in both eyes after one week of treatment, remaining stable while the visual field progressively improved, and was normal in both eyes at one month after initiation of treatment.

## 3 Discussion

At presentation of MOGAD optic neuritis, optic disc swelling is more likely to be bilateral (31–84 %) than in multiple sclerosis and AQ4 optic neuritis.^6^ Furthermore, while rare in cases of both multiple sclerosis and AQ4 optic neuritis, peripapillary hemorrhage is highly suggestive of MOGAD.^7^^,^^8^

Eye pain on eye movements is very common in MS-related optic neuritis and of MOGAD, particularly if inflammation is related to the intra-orbital segment of the optic nerve, while it is uncommon with AQ4 related optic neuritis.^9^^,^^10^

In the context of perioperative period and hyperopia, the diagnosis of NAION should be discussed, whereas the diagnosis of AION must be rapidly ruled out. The relatively preserved VA in the left eye, combined with rapid VA recovery rule out NAION, and the absence of other systemic symptoms, as well as normal FA findings and normal CRP and ESR levels, rule out AION.

In a systematic review and meta-analysis, the incidence of NAION within 2 months post-operative of cataract surgery was almost 100 per 100,000/year, while the spontaneous incidence of NAION was 9 per 100,000/year. In this study, the authors observed that the incidence of post-operative NAION peaked within 72 hours and at 6 weeks, with 73 % of cases occurring within 6 months.^11^ Other studies have confirmed the association between cataract surgery and higher risk of NAION.^2^^,^^12^^,^^13^ Two types of post-cataract surgery optic neuropathy that share clinical characteristics with spontaneous NAION have been described.^14^ The immediate form results from an intra- or perioperative increase of intraocular pressure that leads to a lower perfusion pressure and subsequent ischemia of the optic disc. This form usually occurs hours to days after the surgery. The delayed form occurs some weeks or months after surgery and its pathophysiology is still debated.^14^ In a context of spontaneous NAION or of post-cataract surgery optic neuropathy, the risk of fellow eye involvement at the time of cataract surgery must be discussed with the patient. If the current clinical case is the first presentation of MOGAD occurring a few days after cataract surgery, one may not rule out the possibility that, in a post-operative cataract period, some MOGAD cases may have been previously misdiagnosed as NAION. In this particular context, it is important to rule out other causes of optic disc edema before concluding to post-cataract surgery NAION. Considering AION, there is no mention of any association between AION and post-operative cataract surgery.

In MOGAD, immunotherapies such as rituximab, azathioprine or intravenous immunoglobulins can be used, but they are commonly not used after the first episode. In this case, the use of these therapies would have been debatable, because the risk of recurrence is lower in older individuals.^15^

This case of MOGAD with optic neuritis is atypical by the patient's age at presentation and by its occurrence during the early post-operative period of cataract surgery. However, typical ocular findings and MRI imaging, and the presence of serum MOGAD IgG autoantibodies ultimately led to the diagnosis of MOGAD.

## CRediT authorship contribution statement

**Benjamin Besse:** Writing – original draft, Investigation, Data curation. **Amélie Dos Santos:** Writing – review & editing, Validation. **Mathieu Mille:** Writing – review & editing, Validation. **Francois Bobin:** Writing – review & editing. **Hugo Schultz:** Writing – review & editing, Investigation. **Aline Berthomet:** Writing – review & editing, Validation, Investigation. **Léo Niemerich:** Writing – review & editing, Investigation. **Anna Muszalski:** Writing – review & editing, Investigation. **Favia Gianrocco:** Writing – review & editing, Validation, Investigation. **Nicolas Leveziel:** Writing – review & editing, Validation, Supervision, Investigation, Formal analysis, Conceptualization.

## Authorship

Besse Benjamin: Data curation, investigation, Writing – original draft; Dos Santos Amelie: Data curation, Investigation, Writing – original draft; Mille Mathieu: Validation, Writing – review & editing; Bobin Francois: Validation, Writing – review & editing; Schultz Hugo: Investigation, Writing – review & editing; Berthommet Aline: Investigation, Validation, Writing – review & editing; Niemerich Leo: Acquisition of data, final approval; Muszalski Anna: Acquisition of data, final approval; Gianrocco Favia: acquisition of data, revision, final approval; Leveziel Nicolas: acquisition of data, drafting, conception, design, revision, final approval.

## Ethics approval and consent to participate

This study adhered to the tenets of the Declaration of Helsinki. Written informed consent was obtained from the patient for publication of this case report and any accompanying images.

This case report has never been presented before the submission to American Journal of Ophthalmology Case Reports.

## Funding

None.

## Declaration of competing interest

The authors declare that they have no known competing financial interests or personal relationships that could have appeared to influence the work reported in this paper.
